# COVID-19 Vaccine Perception in Liver Transplant Recipients: Patient-Reported Outcomes and Real-Life Experience from the Bergamo Center

**DOI:** 10.3390/vaccines13050455

**Published:** 2025-04-24

**Authors:** Alessandro Loglio, Elisa Farina, Francesco Ideo, Giovanni Alfieri, Tiziana Negri, Flavia Neri, Valentina Zuccaro, Stefano Fagiuoli, Stefania Camagni, Mauro Viganò

**Affiliations:** 1 Gastroenterology, Hepatology and Transplantation Division, ASST Papa Giovanni XXIII, 24127 Bergamo, Italy; efarina@asst-pg23.it (E.F.); francescoideo@gmail.com (F.I.); sfagiuoli@asst-pg23.it (S.F.); mvigano@asst-pg23.it (M.V.); 2 Gastroenterology, Department of Medicine, University of Milano Bicocca, 20126 Milan, Italy; 3 Associazione Amici del Trapianto di Fegato ODV (Ordine dei Volontari), 24127 Bergamo, Italy; giannyalfieri@gmail.com (G.A.); tinegri@libero.it (T.N.); 4 Department of Organ Failure and Transplantation, ASST Papa Giovanni XXIII, 24127 Bergamo, Italy; fneri@asst-pg23.it (F.N.); scamagni@asst-pg23.it (S.C.); 5 Department of Diagnostic, Paediatric, Clinical and Surgical Science, University of Pavia, 27100 Pavia, Italy; valentina.zuccaro@unipv.it; 6 Department of Infectious Diseases, Fondazione IRCCS Policlinico San Matteo, 27100 Pavia, Italy

**Keywords:** liver transplantation, COVID-19, self-reported outcomes, safety, vaccine, SARS-CoV-2

## Abstract

Background: Bergamo was the most severely affected Italian province at the onset of the 2020 COVID-19 pandemic. The liver transplant (LT) patient population should be among the more sensitized to the concept of health prevention. Long-term data on both perception and outcomes of SARS-CoV-2 vaccination in LT recipients since the COVID-19 vaccine became available in Italy are still lacking. Methods: From May to October 2023, a survey on actively followed LT recipients at our institution was carried on by the local patient’ advocacy (*Associazione Amici del Trapianto di Fegato*) to define the rate of vaccinated subjects, SARS-CoV-2 infections and self-reported COVID-19-related outcomes. Results: Out of the consecutive 753 adult LT recipients invited to the survey, 356 responded (47.3%) [71% male, 63 years old (20–85), LT performed a mean of 9 years (1–26) before vaccination] and were included in the analysis. All patients received the first vaccine dose between December 2020 and January 2022 (81.7% Cominarty^®^, 17.7% Spikevax^®^, 0.3% Vaxzevria^®^ and 0.3% Jcovden^®^). In the following years, adherence to the vaccination policy decreased progressively over time: the second, third, fourth, and fifth vaccine doses were administered to 99%, 94%, 72%, and 22% of the LT population by October, 2023. In total, 43 (12%) and 93 (26%) patients reported a COVID-19 episode before and after [13 (7–21) months] the first vaccination, respectively; none of the LT recipients reported a second COVID-19 infection after the following vaccination cycles. Forty-six (13%) reported short-term post-vaccination mild adverse events but none developed either acute or chronic rejection episodes or hospitalization for COVID-19-related symptoms. A total of 64% of LT recipients resulted positive for anti-nucleocapsid serological test in 2023. Conclusions: COVID-19 vaccines are safe and effective in LT recipients, underlining once again the importance of vaccination in this special population at higher risk of complications from communicable infectious diseases.

## 1. Introduction

The coronavirus disease 2019 (COVID-19) pandemic, caused by severe acute respiratory syndrome coronavirus 2 (SARS-CoV-2), represented the worst pandemic of the last century. SARS-CoV-2 infection carries a high risk of adverse outcomes in patients with specific conditions including chronic liver disease and liver transplant (LT) patients. These patients suffer from immune dysregulation, immunosuppressive state, and associated comorbidities [1,2]. Solid organ transplant (SOT) recipients are at an increased risk of infections due to the immunosuppressant drugs required to prevent graft rejection. In addition, for the same reason, infections can be more severe in transplant recipients than in immunocompetent individuals [3]. Studies on the course and severity of the COVID-19 disease in LT patients reported high hospitalization rates, with more frequent need for mechanical ventilation and high death rates [4,5]. For this reason, vaccinations in LT recipients are highly recommended in LT recipients by the major liver scientific associations such as European (EASL) [3], American (AASLD) [6], and Italian (AISF) [7]. The risk of infection after LT is higher during the first six months, due to the intense immunosuppressive regimens required; therefore, the ideal timing for vaccination should be during the waiting list period: in fact, it is usually advisable to wait 3–6 months after the LT to vaccinate the recipients [8]. Moreover, live attenuated vaccines are contraindicated for LT recipients, due to the risk of develop vaccine-induced infection.

SARS-CoV-2 mRNA-based (Comirnaty^®^ by Pfizer-Bio-NTech, New York, NY, USA, Spikevax^®^ by Moderna, Cambridge, MA, USA) and viral vector-based vaccines (Vaxzevria^®^ by Astrazeneca, Minato City, Janpan, Jcovden^®^ by Johnson & Johnson, New Brunswick, NJ, USA) are approved for the general population, inducing effective protection against COVID-19 [9,10]. Since no data on the efficacy and safety of live-virus vaccines are available among the SOT population, only mRNA vaccines have been recommended in such context. The vaccination campaign has been successful in reducing severity of infections, hospitalizations and deaths from COVID-19 [11].

The COVID-19 vaccination programs in Italy prioritized healthcare workers, cirrhotic and oncologic patients, and transplant recipients, from the end of December 2020. Initially, two doses three weeks apart were recommended (Comirnaty^®^ schedule). The Italian National Health System provided the vaccines at no charge. Subsequently, Health Authorities strongly recommended a booster dose (third dose) about six months after the second (fall, 2021), when restrictions on the free movement of people were still in place to limit the spread of the virus.

Overall, vaccine acceptance has declined since the onset of the pandemic, and less than 70% of the global population has completed the initial COVID-19 vaccination schedule suggested [12]. In Italy, new vaccine doses, given annually (fourth, fifth doses, etc.), were strongly recommended for the entire population, not just for SOT recipients, but were less widely advertised at the national level.

To date, information on the expected duration of vaccine-induced protection, on the requirement for booster vaccinations and on the level of protection against emerging SARS-CoV-2 variants, are still lacking.

For a patient, it is not always easy to express fears and symptoms related to vaccination to a doctor. Underreporting patient symptoms to clinicians has led to the integration of patient-reported outcomes (PROs) in clinical trials to better assess disease symptoms, toxicities, and quality of life, as well as patient experiences and preferences. The Association of Friends of Liver Transplants Recipients of Bergamo (*Associazione Amici del Trapianto di Fegato ODV*—Ordine di Volontari—di Bergamo; http://www.aatf.it; accessed on 19 February 2025) is a volunteer advocacy group founded by LT recipients and their relatives and has been active for over 20 years in Bergamo. The focus of the association is on raising awareness about liver diseases and LT, supporting scientific research, and the development of LT activities. The association also provides logistical and informative support to transplant candidates, recipients, and their families.

Since Bergamo has been the most severely affected Italian province at the onset of the 2020 pandemic, the idea to assess LT patients’ perceptions on COVID-19 infection, vaccination policies, and self-reported symptoms seemed necessary after such catastrophic event. A dedicated survey was promoted by the patients’ advocacy association, integrated with relevant clinical data. The aim of this study was to describe the perception of the vaccination policies for SARS-CoV-2 among LT recipients in one of the areas most afflicted by the COVID-19 pandemic, as well as the safety of the vaccine, as per self-reported outcomes.

## 2. Materials and Methods

### 2.1. Study Design

This is a single-center, real-life, cross-sectional study, based on a survey of COVID-19 vaccination outcomes conducted among adult LT recipients, consecutively enrolled at the liver Transplant Center of ASST Papa Giovanni XXIII Bergamo. The aim of the survey was the evaluation of the perception of mRNA COVID-19 vaccination policy, expressed as number of vaccination cycles performed, safety report and self-reported outcomes.

All 753 patients, transplanted for any indication, aged ≥18 years, alive on 1 March 2023, and attending the outpatient clinic, were invited to complete the survey by the local patients’ advocacy group (The Association of Friends of Liver Transplants). From 15 March to 1 May 2023, the invitations were sent by e-mail, preceded by a phone call from a representative of the advocacy association. The paper version of the anonymous survey was returned either during the planned hepatology follow-up or sent via regular mail. All the responses to the survey, received from 1 May to 31 October 2023, were analyzed for the purpose of this study.

The survey was anonymous, and it included information on the following data:

- patient’s age in 2023;
- year of the LT;
- current weight and height;
- presence of major comorbidities (namely arterial hypertension, diabetes mellitus, dyslipidemia and related therapies);
- any serologically documented episode of COVID-19 (with possible date and hospitalization) before the first vaccine dose;
- dates and number of subsequent SARS-CoV-2 vaccination cycles, together with the definition of possible side effects (date);
- any serologically documented episode of COVID-19 after each vaccine dose (possible date and hospitalization).

Patients could rely on the last hepatological outpatient visit to extract any clinical data.

In addition to the survey, patients were offered the option to submit the results of the latest biochemical tests performed in 2023 and the results of the antibody test (anti-spike and anti-nucleocapsid) conducted in 2023.

SARS-CoV-2 vaccines have been administered by intramuscular injection into the deltoid muscle according to the characteristics of the product and its availability in the local healthcare system during COVID-19 vaccination campaigns (indeed, all LT recipients resident in other Italian provinces underwent the vaccination cycles in the medical area of their reference). All patients maintained their routine post-LT follow-up, with regular outpatient visits in the LT clinic (every three to twelve months according to the time from transplantation).

The following definitions were listed in the description of the survey for the patients, along with dedicated fields to input the main results of the laboratory tests.

- COVID-19 infection was defined by positive result of RT-PCR test or antigenic tests (the tests were performed locally, and they might have differed according to the local laboratory policy).
- Severe COVID-19 was defined as SARS-CoV-2 infection (positive SARS-CoV-2 RT-PCR test) requiring hospitalization and/or causing pneumonia, respiratory failure, sepsis, septic shock, acute respiratory distress syndrome, thrombotic events, myocarditis or death.

### 2.2. Statistical Analysis

The sample size needed to test a significant difference between two groups for a population of 753 patients, with a 5% margin of error and a 95% confidence level (using *p* = 0.5 as the estimated proportion between the two groups) has been calculated to be 255 for our study. Data are displayed as median (range) for continuous variables and as number and percentage for categorical variables. For categorical variables, the Chi-Square statistic was used to assess the statistical significance between groups. Analyses were performed using STATA software (release 10.0, Stata Corporation, College Station, TX, USA).

## 3. Results

### 3.1. Overall

**Figure 1:**
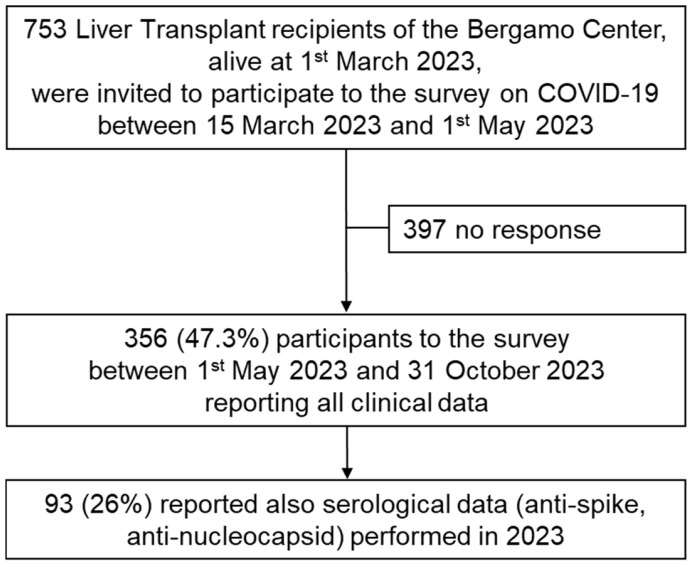
Patient disposition: survey response.

Out of the 753 adult LT recipients invited to participate in the survey, 356 (47.3%) responded positively (Figure 1). In 2023, median age of participants was 63 years old (20–85) and 253 (71%) were male (Table 1). According to the survey, first mRNA vaccination was performed between 27 December 2020 (first date available in Italy) and 1 January 2022. However, 337 (94.7%) LT recipients received their first dose before 31 May 2021. The first dose of vaccine against SARS-CoV-2 was Cominarty^®^ in 291 (81.7%) and Spikevax^®^ in 63 (17.7%); one patient (0.3%) received vector-based Vaxzevria^®^ and one (0.3%) Jcovden^®^. Eight (2.2%) patients did not receive the recommended second injection of the scheduled vaccine program (3, 4 or 12 weeks apart, according to different vaccine). These eight patients did not report in the survey the reason for not receiving the second dose, but they reported no side effects after the first one. Four out of these eight patients received other mRNA vaccine injections 5 months after the first injection, with three of them experiencing a documented COVID-19 infection (without hospitalization) 6 (4–9) months before the first dose.

In Table 1 are represented the self-reported injections of SARS-CoV-2 mRNA vaccine up to 2023: 356 (100%) one dose, 352 (98.9%) two doses, 335 (94.1%) three doses, 256 (71.9%) four doses, and 78 (21.9%) five doses.

### 3.2. Clinical Data

Demographic and clinical features according to the survey are reported in Table 2. Of the 356 patients who participated in the survey, 71% were male, with a median age of 63 (20–85) years at the time of the first SARS-CoV-2 vaccination. The median BMI of the population was 25 (18–45) kg/m^2^, with 58% of the cohort being overweight. The main etiology was viral (57%), with 38% undergoing LT due to hepatocellular carcinoma (HCC). In terms of comorbidities, more than 50% had arterial hypertension, about 34% DM, 34% dyslipidemia, and 38% chronic renal failure. At the time of first vaccination, 44% were taking acetylsalicylic acid, and 30% were on statins. Regarding immunosuppression at the time of the first vaccine administration, 52% were on monotherapy with calcineurin inhibitors (CNI), such as tacrolimus (FK), while the others were on combination therapy with two or more immunosuppressive drugs (Table 2): thirty-nine (11%) patients had experienced acute rejection in the early post-transplant phase, and 17 (5%) had a diagnosis of chronic rejection. Twelve percent of the cohort experienced a documented SARS-CoV-2 infection before receiving the first vaccine dose.

No significant correlation was found between the clinical features of LT patient described in Table 2 and the risk of reported COVID-19 infection either before or after vaccination.

The events following the first dose of the SARS-CoV-2 vaccine up to 2023 are summarized in Table 3. Out of the 356 LT recipients, 93 (26%) reported a documented COVID-19 infection 13 months (7–21) after their first vaccination dose. Importantly, none of these individuals experienced a second symptomatic COVID-19 infection, and none were hospitalized due to COVID-19. Additionally, 43 (12%) patients who had experienced COVID-19 infections before vaccination did not report any subsequent infections after receiving the vaccine. Overall, 136 (38%) of the LT recipients reported having experienced a documented COVID-19 infection at some point, either before or after vaccination.

While the descriptive section on vaccinations, clinical data, and COVID-19 infections was filled out by all patients, the section related to the serological data (anti-spike and anti-nucleocapsid results performed in 2023) was completed by 93 (26%) of the patients. We have observed that the anti-spike titers increased progressively over time with the number of SARS-CoV-2 vaccine doses. Out of these 93 patients, 60 (64%) had experienced a previous SARS-CoV-2 infection (anti-nucleocapsid positive). Anti-spike titers were higher in these 60 (64%) patients who tested positive for the anti-nucleocapsid as compared with the 33 who tested negatives, with levels of 26,667 U/mL (554.2–100,000) vs. 10,418 U/mL (25.6–53,405), respectively.

All the patients that reported COVID-19 infection before their first vaccination (43, 12%) tested positive for anti-nucleocapsid in 2023, with an anti-spike titer of 33,038 U/mL (1910–100,000). None of them experienced a documented COVID-19 infection after first vaccination, and only four (9%) experienced mild AE after first dose of vaccine (diarrhea, asthenia).

A minority of the cohort reported adverse events (AE) after vaccine administration (13%); all symptoms were mild and self-limited. None developed acute rejections, an increase in Aminotransferase level, hospitalization or death after vaccine inoculation. It is relevant that, despite reported mild AE, these patients received 4 (3–5) doses of COVID-19 vaccine.

Overall, the immunosuppressive regimens were modified in 11 patients (3%) in 2023 compared to 2021; this event was not related to vaccination nor COVID-19 infection, with a small increase of everolimus use.

Table 4 shows the trend of the anti-spike titer in 43 patients, for whom more than one blood sample is available at different time points, from after the first two doses of the vaccine to the last available sample collected in 2023, along with the anti-nucleocapsid titer. Additionally, the number of vaccinations, as well as COVID-19 infection before and after the first vaccine dose, are reported, as indicated in the survey. It can be observed that, in almost all transplanted patients, the anti-spike titer increases over time and with the number of vaccinations. Due to the limited number of antibodies reported in the survey and the fact that not all tests were conducted in the same laboratory, it was not possible to identify a protective antibody level against COVID-19. However, as reported in the literature, it appears that the higher the antibody titer, the greater the protection against the infection, and that new vaccinations are safe and lead to an increase in the antibody titer. Patients who had a previous COVID-19 infection developed higher antibody titers, and these titers increased with each subsequent vaccination. Our study showed that patients received multiple vaccinations without reporting new adverse events, and with a reduction in the infection rate (no new COVID-19 infections were reported after the first post-vaccination event).

## 4. Discussion

In Italy, the COVID-19 vaccination became available and recommended for SOT recipients at the end of December 2020. Initially, two doses three weeks apart, followed by a suggested third dose six months later were indicated (fall 2021) (Comirnaty^®^ schedule), when restrictions on people’s movements were still in place to limit the spread of the virus. New vaccine doses, given annually (fourth, fifth doses, etc.), were strongly recommended for the entire population, including SOT recipients, but were less widely advertised at the national level. However, locally, both health personnel and the patients’ advocacy association continued to recommend SARS-CoV-2 booster shots.

The first result of this study is that only 47.3% of the adult LT population who were followed in Bergamo responded to the survey promoted by the patients’ advocacy group. This is an important point to be noticed in a population that is expected to be highly loyal to the transplant reference center. This could be explained by the patients’ limited interest in the issue of vaccines, and particularly COVID-19, which has been the source of aggressive general discussion in those years. Additionally, it must be noted that, since several LT recipients reside outside the province of Bergamo and even the Lombardy region, it is likely that they might have been less emotionally exposed to the initial COVID-19 pandemic and therefore less sensitized to the topic. Due to the design of the study and the anonymous survey proposed by the patient advocacy group, it was not possible to clinically and socially differentiate the patients who responded to the survey from the patients that did not. This represents an unavoidable clear limitation of the study; however, it also reflects patients’ adherence to COVID-related initiatives and vaccination efforts. In any case, the survey reached almost half of the transplant population, which, based on the analysis of the questionnaires received, seems to be adequately representative of the liver transplanted population in Bergamo.

This adherence rate to the surveys is similar to a previously published study. An anonymous web-based questionnaire was conducted in adult Chinese renal transplant recipients in May 2021: 813 respondents from 30 provinces all over China, participated in the survey, with a response rate of 40.0%. Among the respondents, none of them had a history of SARS-CoV-2 infection. Only 5.7% of the respondents had received any COVID-19 vaccine, while 94.3% had none; 22.8% SOTRs reported that they were willing to get vaccinated, while 65.6% declared that they were still hesitant and 11.6% refused any vaccine [13]. In the specific case of mRNA vaccines, patients were concerned mainly that they were developed and approved rapidly and that there was lack of knowledge regarding long-term effects and the potential presence of toxic substances within the vaccine [14,15,16].

The vaccination adherence rate progressively declined over time, especially when looking at the 356 patients who responded to the survey. Vaccination adherence remained high for the first doses and during lockdown periods, when movement was restricted and the vaccination certificate was required for greater freedom of movement. As highlighted in Table 1, adherence to the fifth dose was very low, with only 21.9% of patients reporting having received it (in comparison to the 98.9–100% adherence reported for the first two doses).

According to the survey, we were able to observe that none of the 356 patients reported severe adverse reactions to vaccine, required hospitalization, or experienced acute rejection after SARS-CoV-2 vaccine inoculation. Of note, being a survey on alive patients only, any event related to the deceased population cannot be analyzed in this specific cohort. This survey aside, it must be noted that in our clinical practice at the LT center, severe adverse events to vaccine inoculation were not observed nor reported. The data of “no hospitalization” emerging from the survey, indeed correspond to a relevant safety key point, and generally it can be stated that vaccination was well-tolerated and safe. Only a minority of patients reported adverse events, mild and quite “expected” (mild fever, asthenia) in all. Therefore, this could be considered a confirmation of the safety of the new mRNA vaccine. Even the two patients who received the first vaccination with a viral vector did not report any adverse events or rejection. Moreover, even patients experiencing mild AE after the first dose did not report AE after the subsequent new doses of vaccine, thus defining the relevance of the vaccination against new SARS-CoV2 variants.

No significant correlations were found between the characteristics of LT recipients and the risk of experiencing a COVID-19 infection, as reported in the survey. However, several patients might have had a paucisymptomatic infection, without undergoing any specific testing. The availability of serological test only from patients with symptomatic disease and not from the whole cohort represents a further limitation of our study, even though this was not the specific objective of the survey. Moreover, as shown in other paper on this field, no clear risk factors for COVID-19 infection in LT recipients were identified [17,18].

Another important result from this survey is the percentage of reported COVID-19 infection in LT recipients: 12% before and 26% after vaccination, 38% in total. Moreover, according to serological data available in 26% of this cohort, 64% of LT recipients followed by our LT center in Bergamo, resulted positive for anti-nucleocapsid in 2023. One could speculate that vaccinations made infection less symptomatic, given this difference in serological and clinical aspect of the disease: the percentage of transplant patients who had a confirmed prior exposure to COVID-19 (64% anti-nucleocapsid positive in 2023) is higher than the 38% of patients who overall reported a COVID-19 infection (symptomatic and therefore underwent a confirmatory serological test). Additionally, the trend of anti-spike titers increased post-vaccination, rising over time with repeated doses, and was higher in patients that experienced COVID-19 before first vaccination. None of our patients experienced a second COVID-19 infection after the first dose of vaccine, likely as the result of the progressively increasing antibody titers, along with a likely lower virulence of the virus in 2022 and 2023. Of note, even if serological data are available only in 93 LT patients, a rate of 64% anti-nucleocapsid positivity is higher than a 51.8% from a recent Spanish single-center LT cohort [19]. A survey conducted on 251 pediatric liver transplant patients at the end of 2022, where RT-PCR data were available, revealed an Omicron infection rate of 56.2% (141/251), which is similar to the rate reported in the general population (around 60%). Furthermore, in that study, only 61.6% of parents were in favor of COVID-19 vaccination despite the Omicron outbreak [20]. This confirms the consistency of the data emerging from our survey, with 64% of COVID-19 infections in the adult population, despite the aforementioned limitations. Unfortunately, it also confirms a global trend of reduced adherence to vaccination as we move further from the pandemic. This data on adherence could also be influenced by the response rate to the survey, since only half of the patients followed in Bergamo responded to the questionnaire. Those who did respond may also well be the ones generally more compliant with healthcare providers’ recommendations.

Another limitation of this study is related to the fact that serological and virological test were not standardized nor centralized to a single laboratory, but vary according to local laboratory availability, since most patients reside outside the province or region. However, it must be noted that usually, patients tended to consistently use the same laboratory for antibody testing, and therefore, the observed increase in the anti-spike titer over time for each individual patient is valid, supporting the correlation with the increase in the number of vaccinations.

Our study shows that patients received multiple vaccinations without reporting new adverse events, and with a reduction in the infection rates (no new COVID-19 infections were reported after the first post-vaccination event). Given the constant presence of viral mutations, even though cross-protection from the vaccine seems to be present, an annual COVID-19 vaccination program is advisable.

The main limitation of this study, besides being a survey, is the lack of complete serological data for the entire population. On the other hand, it allows us to explore the level of perception of the importance and acceptance of vaccination in a highly loyal and medically engaged population in a region heavily affected by the outbreak of the COVID-19 pandemic. No difference in the etiology of liver disease or between male and female patients was highlighted in relation to the completion of the survey.

To overcome the limitations of this study, a serological test for anti-spike and anti-nucleocapsid antibodies should be performed in the same laboratory for all liver transplant patients. Additionally, two questionnaires should be administered: one anonymous and one administered by a healthcare provider, to assess different responses. The results of the serological test should be matched with the questionnaire administered by the healthcare provider and the clinical characteristics of the patient.

While aiming for new additional data, from our experience, the strong emotional impact of the actual risk of death related to severe COVID-19 in 2020, as well as the desire for mobility in the spring of 2021 (vaccination equaling freedom of movement), resulted in nearly 100% vaccination coverage. In the subsequent waves of SARS-CoV-2, as mortality decreased and the perceived risk of morbidity and mortality from COVID lessened, adherence to vaccination recommendations significantly declined.

## 5. Conclusions

The mRNA vaccine remains safe and effective in LT recipients. This survey, conducted 2.5 years after the first SARS-CoV-2 vaccination in the transplant patient population, showed that the rate of interest on this topic declined (47.3% adherence to the questionnaires; 22% reported fifth dose of vaccine). It also demonstrates that COVID-19 vaccines are safe (with 13% reporting only mild adverse events) and that the low number of reported events is likely due to repeated vaccination cycles with new doses targeting new variants (64% of patients had contracted COVID-19, based on serological data in 2023). New communication strategies are needed to raise awareness among SOT patients about the importance and utility of administering periodic, updated vaccinations against new variants, as they can further boost antibody levels, enhancing protection against emerging strains of the virus.

## Figures and Tables

**Table 1:** Age, gender, and number of COVID-19 vaccine doses received by the 356 liver transplant recipients. Data reported by patients in surveys collected from May to October 2023.

Variables	N = 356
Age at first COVID-19 vaccination, years *	63 (20–85)
Males	253 (71%)
Vaccine doses received up to 2023	
One °	356 (100%)
Two	352 (98.8%)
Three	335 (94.1%)
Four	256 (71.9%)
Five	78 (21.9%)

* median (range); °: 291 (81.7%) Cominarty, 63 (17.7%) Spikevax, 1 (0.3%) Vaxviera, 1 (0.3%) Jcovden.

**Table 2:** Demographic and clinical characteristics of 356 subjects at the first dose of SARS-CoV-2 vaccine.

Variables	N = 356	Variables	N = 356
Age at LT, years *	55 (0–68)	Diabetes	131 (37%)
Age at first vaccination, years *	63 (20–85)	Arterial Hypertension	206 (58%)
Time from LT to vaccine, yrs *	9 (1–26)	Dyslipidemia	121 (34%)
Males	253 (71%)	Chronic renal failure	135 (38%)
BMI, kg/m^2^ *	25 (18–45)	Cardioaspirin	157 (44%)
Overweight, BMI > 25 kg/m^2^	206 (58%)	Statin	107 (30%)
Etiology		Immunosuppres. Regimen ^@^	
Viral (HBV/HDV/HCV) °	203 (57%)	FK	186 (52%)
Metabolic/alcoholic	60 (17%)	FK + MMF	103 (29%)
Immuno-mediated (AIH, PSC, PBC) °°	36 (10%)	FK + EVL	32 (9%)
Polycystic liver	21 (6%)	FK + Steroid	14 (4%)
Others °°°	36 (10%)	CSA + MMF or steroids	14 (4%)
		Others °°°°	7 (2%)
Hepatocellular carcinoma at LT	135 (38%)	Speen size at first dose, cm *	10 (9–23)
Kidney transplant	14 (4%)	Numbers of acute rejection before first vaccine dose ^#^	39 (11%)
Re-transplant	14 (4%)	Numbers of chronic rejection before first vaccine	17 (5%)
Smoke Active/Previous	21 (6%)/39 (11%)	SARS-Cov2 infection before the first vaccine dose	43 (12%)

* median (range); ° 61 HBV, hepatitis B virus; 96 HCV, hepatitis C virus; 46 HDV, hepatitis Delta virus; °° 7 AIH, autoimmune hepatitis; 16 PSC, primary sclerosing cholangitis; 13 PBC, primary biliary cholangitis; °°° hepatoportal sclerosis, idiopathic liver fibrosis, Caroli disease. ^@^ FK, tacrolimus; MMF, Mycophenolate mofetil; EVL, everolimus; CSA, cyclosporine; °°°° sirolimus or MMF monotherapy. LT, liver transplant. ^#^ time from rejection to first vaccine: 9.1 (2.8–22.1) years.

**Table 3:** Events after first vaccine dose, up to 2023.

Variables	N = 356
Time from first vaccine to survey, years *	2.5 (2.1–2.8)
Any adverse events reported after vaccine	46 (13%) °
Numbers of acute rejections	0
Numbers with first COVID infections reported after vaccine	93 (26%)
Time from first vaccine to COVID, months *	13 (7–21)
Numbers with second COVID infections reported after vaccine	0
Numbers of hospitalization for COVID	0
Numbers of deaths	0
Immunosuppression regimen at last visit ^@^	
FK	171 (48%)
FK + MMF	107 (30%)
FK + EVL	25 (7%)
FK + Steroid	7 (2%)
CSA + MMF or steroids	10 (3%)
EVL + MMF	18 (5%)
Others °°	18 (5%)
Anti-spike titers **	
June 2021	564 (3.8–33,789) U/mL
December 2021	1483 (61–8606) U/mL
Summer 2023	11,360 (6.6–100,000) U/mL
Anti-nucleocapsid positivity in 2023 **	60 (64%)

* median (range). ° 23 (50%) asthenia, 3 (7%) diarrhea, 4 (9%) fever, 3 (7%) labial edema, 4 (9%) itching, 4 (9%) myalgias, 4 (9%) headache. °° sirolimus or MMF monotherapy. ** available in 93 patients. ^@^ FK, tacrolimus; MMF, Mycophenolate mofetil; EVL, everolimus; CSA, cyclosporine.

**Table 4:** Changes in anti-spike titers in patients with reported levels, according to previous COVID-19 infection, number of vaccinations, COVID-19 infection after first vaccine dose and related time, and anti-nucleocapsid in 2023 (43 available samples).

Patient ID	COVID-19 Infection Before First Vaccination	Number of COVID-19 Vaccinations	Anti-Spike Titer, After Second Vaccine Dose	Anti-Spike Titer, After Third Vaccine Dose	Anti-Spike Titer, After Fourth Vaccine Dose	Anti-Spike Titer, Last Evaluation	First COVID-19 Infection After First Dose of Vaccine	Months Between First Vaccine and First COVID-19	Anti-Nucleocapsid Positive in 2023
2	No	5	80.29	24.9	942	22,139	No		No
3	No	4	4330			4330	No		
4	No	4	736			150	No		
5	No	5	5000	5000		5000	No		Yes
12	No	5	342			12,300	No		
14	No	4	564			4351	Yes	13	
15	No	4	120			12,560	No		
16	No	5	13.9			18,985	No		
18	No	4	520			40,000	Yes	13	
20	Yes	4	1747	1483		28,463	No		Yes
21	Yes	4	150			100,000	No		Yes
23	Yes	5	52	8606		1960	No		
24	No	5	5010			26,667	No		Yes
26	No	4	1357			150	Yes	13	
33	No	4	5113	4347		75,342	Yes	10	Yes
34	No	3	134			5000	No		Yes
36	No	4	74	1057		2006	No		No
37	No	4	3.8			12,492	No		
39	No	5	30.12			17,262	No		Yes
40	No	3	108			15,137	No		Yes
42	No	5	37.8	507		33,527	No		No
44	No	5	5052			3970	No		
45	No	3	5.6			7804	No		Yes
46	No	4	0.03	61,4		13,700	Yes	21	
49	Yes	5	434	927	1132	49,648	No		Yes
50	No	4	157			18,282	Yes	12	Yes
51	No	4	73	1800		3152	No		Yes
52	No	4	1.85	454		43,075	No		No
55	No	4	186	5680		5680	No		No
61	No	3	1710			2948	No		
65	No	5	1883	229	4054	31,038	No		Yes
66	No	3	1226	983		10,418	No		No
67	No	4	33,789			11,237	No		Yes
69	No	4	65.40	2443		47,152	Yes	16	No
72	No	3	627			40,000	Yes	19	
73	No	3	928			8629	No		Yes
74	No	3	2080	2080		36,846	No		Yes
75	Yes	3	380			60,952	No		Yes
79	No	3	21			629	Yes	18	
80	No	4	1198			2080	Yes	12	
84	No	3	4963			1778	Yes	15	Yes
88	No	4	25.25			29,500	No		No
90	No	4	9.6			2080	No		

## Data Availability

Data from the present study are kept confidential but can be provided upon reasonable request to the authors.
